# Improvements of sensorimotor processes during action cascading associated with changes in sensory processing architecture–insights from sensory deprivation

**DOI:** 10.1038/srep28259

**Published:** 2016-06-20

**Authors:** Krutika Gohil, Anja Hahne, Christian Beste

**Affiliations:** 1 Cognitive Neurophysiology, Department of Child and Adolescent Psychiatry, Faculty of Medicine of the TU Dresden, Germany; 2 Saxonian Cochlear Implant Center, Division of Phoniatrics and Audiology, Department of Otorhinolaryngology, Faculty of Medicine of the TU Dresden, Germany

## Abstract

In most everyday situations sensorimotor processes are quite complex because situations often require to carry out several actions in a specific temporal order; i.e. one has to cascade different actions. While it is known that changes to stimuli affect action cascading mechanisms, it is unknown whether action cascading changes when sensory stimuli are not manipulated, but the neural architecture to process these stimuli is altered. In the current study we test this hypothesis using prelingually deaf subjects as a model to answer this question. We use a system neurophysiological approach using event-related potentials (ERPs) and source localization techniques. We show that prelingually deaf subjects show improvements in action cascading. However, this improvement is most likely not due to changes at the perceptual (P1-ERP) and attentional processing level (N1-ERP), but due to changes at the response selection level (P3-ERP). It seems that the temporo-parietal junction (TPJ) is important for these effects to occur, because the TPJ comprises overlapping networks important for the processing of sensory information and the selection of responses. Sensory deprivation thus affects cognitive processes downstream of sensory processing and only these seem to be important for behavioral improvements in situations requiring complex sensorimotor processes and action cascading.

In most everyday situations sensorimotor processes are quite complex because situations often require execution of several actions in a specific temporal order. For this reason, we heavily depend on action cascading, which is defined as the ability to generate, process, and execute separate task goals and responses in an expedient temporal order to produce an efficient goal-directed multi-component behavior^1^,^2^,^3^,^4^. For example, when driving a car it is usually easy to drive around the corner and follow the street. However, sometimes you drive around corner and suddenly notice a road construction zone, which was not indicated before you turned your car around the corner. You will then have to stop your car and turn it into another direction. This example shows that to achieve a goal it is sometime necessary to stop/interrupt ongoing behavior or a response and immediately change to another response (option); i.e. you have to cascade different actions to achieve a task goal. Action cascading therefore refers to executive control processes (e.g. stopping/inhibition processes and switching processes) that are put in a close temporal order. To examine such processes, stop-change paradigms are frequently used.

During action cascading, examined using stop-change tasks, usually different sensory modalities have to be integrated to process stop and change stimuli and therefore action cascading requires attentional shifting^3^,^5^,^6^,^7^,^8^,^9^. Sensory processing and the number of sensory modalities that need to be integrated have thus been shown to play a major role in above processes^10^,^11^. However, attentional selection processes and response selection processes are less demanded when action cascading processes are triggered by uni-modal stimuli, compared to bi-modal stimuli^12^. While these results show that manipulations of the complexity of sensory information have an effect on action cascading processes, it remains elusive if changes in the neural architecture to process sensory stimuli also have an effect on action cascading processes. In other words: Are action cascading processes modulated when sensory stimuli are not changed, but the neural architecture to process these stimuli is altered? In fact, this should be the case because changes to the processing architecture in one module of a network important for control likely change the global processing workspace required to cope with cognitive tasks^13^.

Such changes in neural architecture that may affect effortful sensorimotor processing may result from early sensory deprivation. Early sensory deprivation can induce changes in deprived sensory cortices to enable processing of stimuli from intact sensory modalities^14^. These cross-modal take-over, or reorganization processes have, for example, been described in deaf subjects for the visual modality^15^,^16^,^17^,^18^. Subjects with (prelingual) deafness may therefore serve as a model to examine in how far changes in the neural architecture to process sensory stimuli have effects on action cascading processes. Interestingly, it has conclusively been shown that cross-modal reorganization processes are associated with superior perceptual performance in the intact sensory modality^19^,^20^,^21^,^22^. An intriguing hypothesis that follows from this is that action cascading processes may also be more efficient or more accurate when neural architecture of the intact sensory system is reorganized to compensate for the deprived sensory system. However, this would imply that improvements are not restricted to the sensory processing stage but may affect downstream processing stages of response selection as well. This is likely, since previous results suggest that altered perceptual and attentional processes have an effect on action cascading at the response selection level^12^,^23^. This seems all the more plausible, since response selection processes during action cascading have been shown to depend on the activity in the temporo-parietal junction (TPJ)^3^,^8^, which is also involved in sensory integration processes^24^,^25^. Moreover, concerning the possible importance of a global processing workspace^13^ a change in one network module has effects on every process mediated by the global processing workspace and thus also on response selection processes.

In the current study, we test this hypothesis using prelingually deaf subjects as a model. We test this hypothesis using a system neurophysiological approach, combining EEG and source localization techniques to delineate the functional neuroanatomical network, which is differentially modulated between prelingually deaf subjects and controls during action cascading. Action cascading is examined in a stop-change paradigm. During this task a response had to be stopped (interrupted) in 33% of trials and an alternative response (i.e. change response) had to be executed. Stopping and changing processes were signaled by visual stimuli. To modulate the complexity of action cascading, and especially of the change of a response, we varied the time interval between the STOP and the CHANGE stimuli (i.e. stop-change delay, SCD). The CHANGE stimuli were either presented at the time as the STOP stimuli, or 300 ms thereafter.

Concerning the effect in the EEG data, the P1 has been suggested to be a measure of perceptual and attentional gating processes^26^, while the N1 is thought to reflect a top-down guided discrimination process which selectively allocates attention to relevant stimulus features^27^,^28^,^29^. Given that, there is superior perceptual performance in the intact sensory modality, processes reflected by the P1 and N1 should be more efficient in the patients with pre-lingual deafness and may be therefore reduced in the amplitude. Response selection processes are reflected by the P3 event-related potential^3^,^30^. However, if changes associated with prelingual deafness affect response selection, we expect to find modulations of the P3 ERP as well. In the task applied, more efficient action cascading has been associated with a reduced P3^3^,^31^. The reason is that in the stop-change paradigm was applied the P3 reflects inhibition processes and changing processes. This is especially critical in the SCD0 condition where both processes likely occur at the same time. This most probably yields a strong interference between the STOP and CHANGE task goals at a strategic response selection bottleneck^32^. It is therefore possible that inhibitory control processes needed to manage the stopping and changing of responses are intensified. Such an intensification of response inhibition efforts has frequently been shown to be related to higher P3 amplitudes^33^ and may have led to the repeatedly found effect that a stronger P3 was related to poor task performance in the task applied^3^ (e.g. Mückschel *et al*.^3^). We therefore expect response selection processes (reflected by the P3) to be more efficient and accurate in prelingually deaf subjects and that these effects are associated with modulations in fronto-parietal regions.

## Results

### Behavioral data

The descriptive behavioral data are shown in supplementary Table S1. The analysis of the reaction times (RTs) on GO trials revealed main effect of “group” (F(1,25) = 26.30; p < 0.001; η_p_^2^ = 0.513). RTs of prelingually deaf patients were longer (703 ms ± 65) than of control participants (519 ms ± 67). A mixed effect ANOVA using the within-subject factors “SCD interval” and the between-subject factor “group” was run to analyze the RTs data on the CHANGE stimulus. There was a significant main effect of “group” (F(1,25) = 11.72; p = 0.002; η_p_^2^ = 0.319) showing that the prelingually deaf patients were slower (855 ms ± 42) than the control group (643 ms ± 44). There was also a main effect of “SCD interval” (F(1,25) = 176.60; p < 0.001; η_p_^2^ = 0.876) indicating that the participants were generally slower in the SCD0 condition (830 ms ± 31) than the SCD300 condition (668 ms ± 31). However, there was no interaction of “SCD interval × group” (F(1,25) = 0.18; p = 0.673), which indicates that there were no differential effects of groups on RTs in the two SCD conditions.

**Figure 1:**
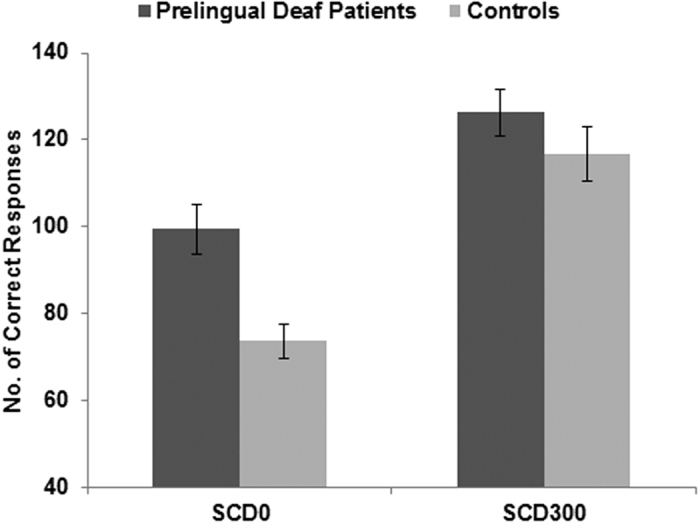
Results for the accuracy data (correct responses). The plot shows that the group differences were larger in the SCD0 than in the SCD300 condition.

A mixed effect ANOVA using the within-subject factors “SCD interval” and the between-subject factor “group” was run to analyze the accuracy (i.e., the absolute frequency of correct responses) on the CHANGE stimulus. There was a main effect of “SCD interval” (F(1,25) = 137.72; p < 0.001; η_p_^2^ = 0.846) showing that participants were generally more accurate in the SCD300 condition (121.52 ± 4.08) than the SCD0 condition (86.47 ± 3.49). There was also a main effect of “group” (F(1,25) = 6.39; p = 0.018; η_p_^2^ = 0.204), showing that the prelingually deaf patients were more accurate (112.82 ± 4.84) than the control group (95.17 ± 5.03). Importantly, there was an interaction of “SCD interval × group” (F(1,25) = 7.39; p = 0.012; η_p_^2^ = 0.228), indicating differential effects of SCD interval in the prelingually deaf group and the control group. This interaction is shown in Fig. 1.

Post-hoc independent samples t-tests were used to examine the interaction in more detail. These revealed that group differences were evident in the SCD0 condition (t(25) = 3.69; p = 0.001), which is the difficult condition because both STOP and CHANGE stimuli are presented at the same time to the participants. However, the group differences were not significant in the SCD300 condition (t(25) = 1.16; p = 0.254). In terms of accuracy, there was no group effect on GO trials (F(1,25) = 3.52; p = 0.072). For SC trials, the staircase procedure was applied to access SSRTs so the accuracy for the STOP response cannot differ. The analysis of SSRTs revealed a main effect of “group” (F(1,25) = 23.27; p < 0.001; η_p_^2^ = 0.482) indicating that the mean stop signal reaction time was higher in the prelingually deaf patients (882 ms ± 62) than in the control participants (444 ms ± 65). For the behavioural data, the inclusion of “age” as covariate did not change the pattern of results (all F < 0.33; p > 0.3).

To examine whether the duration of cochlear implantation has an effects on the behavioral effects, Pearson’s bivariate correlations were calculated. This was therefore done for the behavioral effects in the SCD0 condition and the SSRT, but revealed no correlation (all r < −0.343, p > 0.23).

Summarizing the behavioral data, we found that the prelingually deaf patients had prolonged RTs as well as more accuracy in response selection in case of simultaneous inputs. However, a speed-accuracy trade off can be ruled out because the RTs did not show a differential modulation across SCD conditions and groups, as it was found for the accuracy data. Prelingually deaf patients therefore show better performance in action cascading in terms of accuracy.

### Neurophysiological data

#### P1 and N1

**Figure 2:**
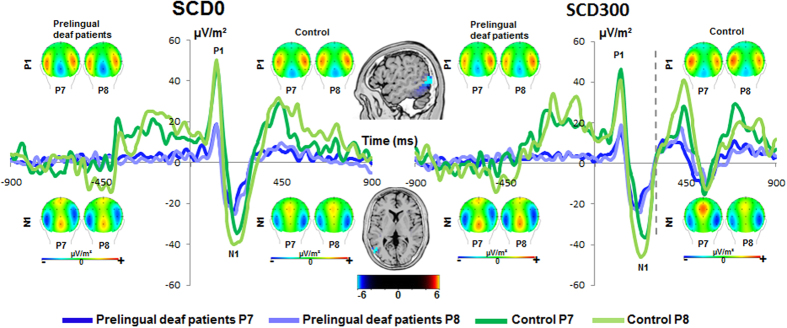
The P1 and N1 ERPs are shown. Time point 0 denotes the time point of STOP stimulus presentation. For the SCD300 condition (right figure part) the vertical dashed line denotes the time point of CHANGE stimulus presentation. In the SCD0 condition (on the left), the CHANGE stimulus was presented at the same time with the STOP. The visual P1 and N1 elicited by the visual CHANGE stimulus in the both groups are shown for electrodes P7 and P8 for the control and prelingually deaf group (refer figure for details). The scalp topography plots show typical maps for the P1 and N1 ERPs. The sLORETA source localization revealed that the peak amplitude differences in the P1 ERP between groups was due to the activation differences in the middle occipital gyrus (BA19).

The ERPs on the P1 and N1 are shown in Fig. 2.

The P1 amplitudes were analyzed in a mixed effect ANOVA using the factors “SCD interval” and “electrode”, as within-subject factors and “group” as between-subject factor for the STOP and CHANGE stimuli. For the STOP stimulus P1 amplitudes showed no main effects of SCD interval and electrodes (all F < 1.7; p > 0.2). Moreover, there were no interactions of SCD interval × group, of electrodes × group, of SCD interval × electrodes, of SCD interval × electrodes × group (all F < 1.54; p > 0.2). There was a main effect of “group” (F(1,25) = 10.16; p = 0.004; η_p_^2^ = 0.289) indicating that the STOP-P1 was smaller in prelingually deaf patients (14.54 μV/m^2^ ± 4.66) than in controls (35.97 μV/m^2^ ± 4.84). The sLORETA analysis suggests that this difference was due to activity changes in the middle occipital gyrus (BA19).

For the CHANGE stimulus P1 amplitudes revealed a main effect of the “SCD interval” (F(1,25) = 20.22; p < 0.001; η_p_^2^ = 0.447) indicating that P1 was larger in the SCD0 condition (−22.05 μV/m^2^ ± 4.02) than the SCD300 condition (−7.35 μV/m^2^ ± 2.15). There was no main effect of the “electrodes” and “group” (F < 2; p > 0.05). Moreover, there was no interaction of “electrodes × group” and of “SCD interval × electrodes × group” (F < 3; p > 0.05). There was an interaction of “SCD interval × electrodes” (F(1,25) = 7.42; p = 0.01; η_p_^2^ = 0.229). Further analysis of the interaction in the post-hoc test revealed that the difference between the electrodes was significant in the SCD0 condition (t(25) = −3.03; p = 0.006), but not in the SCD300 condition (t(25) = −1.05; p = 0.3).

The N1 amplitude was analyzed with the same kind of mixed effects ANOVA; i.e. for the STOP and the CHANGE stimuli. For the STOP stimuli, N1 amplitude revealed a main effect of “SCD interval” (F(1,25) = 10.84; p = 0.003; η_p_^2^ = 0.302) showing that the N1 was larger (i.e. more negative) in the SCD0 condition (−22.05 μV/m^2^ ± 4.02) than the SCD300 condition (−15.21 μV/m^2^ ± 2.96). There was a main effect of “electrodes” (F(1,25) = 7.866; p = 0.01; η_p_^2^ = 0.239) showing that N1 was more negative in the P8 electrode (−23.09 μV/m^2^ ± 3.9) than in the P7 electrode (−14.17 μV/m^2^ ± 3.55). There was no significant main effect of “group” (F(1,25) = 0.103; p = 0.75). There were no interactions of SCD interval × group, of electrodes × group, of SCD interval × electrodes, or of SCD interval × electrodes × group (all F < 0.14; p > 0.5) found for the N1 ERP.

For the CHANGE stimuli, N1 amplitude revealed a main effect of “SCD interval” (F(1,25) = 20.23; p < 0.001; η_p_^2^ = 0.447) showing that the N1 was larger (i.e. more negative) in the SCD0 condition (−22.05 μV/m^2^ ± 4.02) than the SCD300 condition (−7.35 μV/m^2^ ± 2.15). There was no main effect of “electrodes” (F(1,25) = 1.09; p = 0.3). There was no significant main effect of “group” (F(1,25) = 8.27; p = 0.92). There were no interactions of SCD interval × group, of electrodes × group, or of SCD interval × electrodes × group (all F < 3; p > 0.5) found for the N1 ERP. There was an interaction of “SCD interval × electrodes” (F(1,25) = 7.42; p = 0.01; η_p_^2^ = 0.229), which did not withstood bonferroni-corrected post-hoc testing (t(25) < 0.71; p > 0.2). It may be argued that the baseline used biases the effects. However, also when using a baseline from −200 to 0 (i.e. locking point), or when using a peak-to-peak quantification method of the ERPs, which is baseline independent, the pattern of results (i.e. effects between groups) remained the same.

Summing up the findings on ERP components related to perceptual gating and attentional selection, we found that P1 and N1 ERPs displayed differential effects. While the P1 ERP was differentially modulated for the CHANGE stimulus in the SCD conditions, the N1 ERP was differentially modulated for the both STOP and CHANGE stimuli in the SCD conditions. However, the direction of the differences remained same in the both the cases (i.e. larger in the SCD0 condition than the SCD300 condition) (e.g.^12^. Groups only differed in terms of perceptual gating (P1), however, not different for the SCD conditions. The analysis of the SNR-data revealed no main or interaction effects (all F < 1.05; p > 0.3), showing that the results obtained are unbiased with respect the SNR. Moreover, the inclusion of “age” as covariate did not change the pattern of results (all F < 0.21; p > 0.3).

#### P3

**Figure 3:**
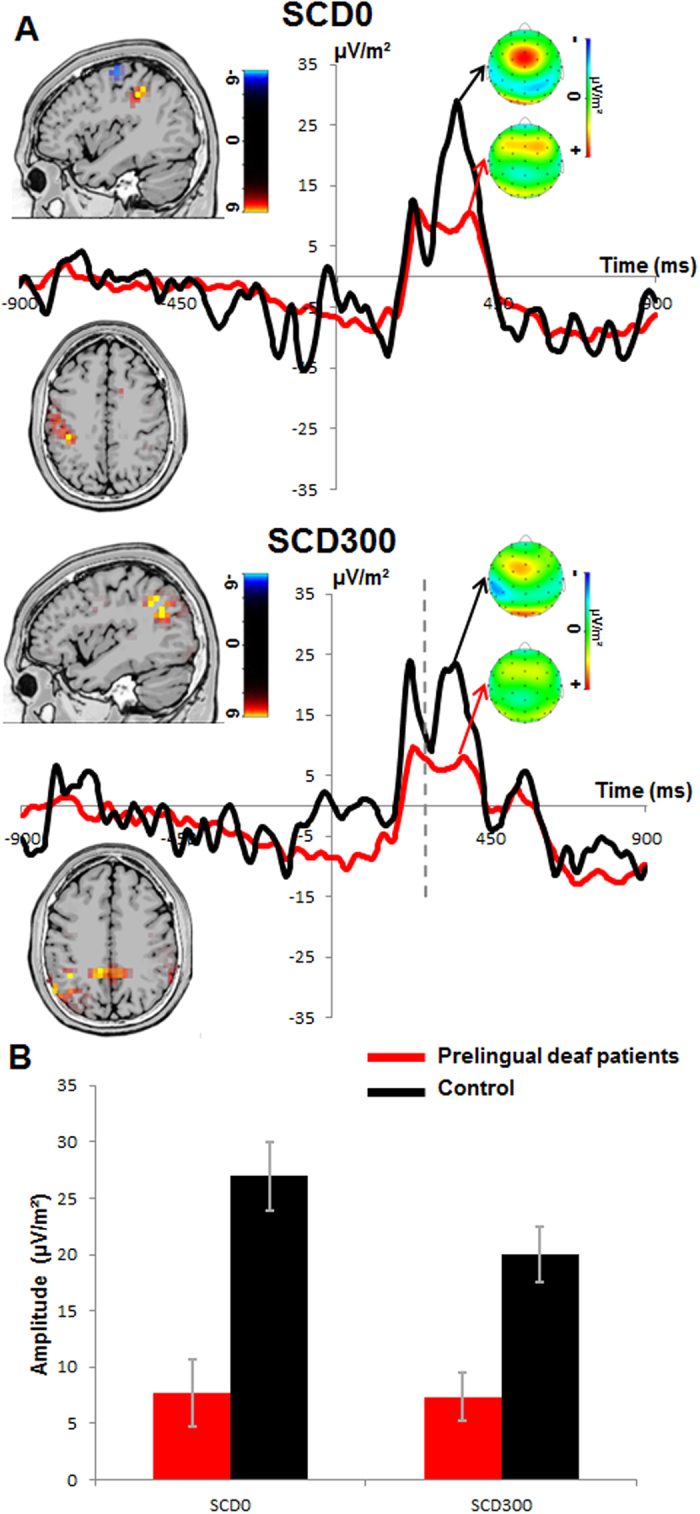
(**A**) The P3 ERPs are shown. Time point 0 denotes the time point of STOP stimulus presentation. Patients in the prelingually deaf group are shown in red curves, controls in black curves for electrode Cz. For the SCD300 condition (middle figure part) the vertical dashed line denotes the time point of CHANGE stimulus presentation. In the SCD0 condition (on the left), the CHANGE stimulus was presented at the same time with the STOP. The scalp topography plots show typical maps for the P3 ERPs in the paradigm applied. sLORETA source localization revealed that group differences in P3 amplitudes were due to a higher activation of the TPJ in the control group. (**B**) A bar graph depicting the interaction and the modulation of the P3 peaks in the different groups (prelingually deaf patients and control) across SCD conditions. The graph shows that the P3 peak was larger in the control group in both SCD conditions than the prelingually deaf patients.

The P3 at electrode Cz is shown in Fig. 3.

The mixed effects ANOVA using the factors “SCD interval” as within-subject factors and “group” as between-subject factor revealed a main effect of “SCD interval” (F(1,25) = 4.727; p < 0.05; η_p_^2^ = 0.159), showing that the P3 was larger in the SCD0 (17.32 μV/m^2^ ± 2.1) than in the SCD300 condition (13.71 μV/m^2^ ± 1.6). There was a significant main effect of “group” (F(1,25) = 22.158; p < 0.001; η_p_^2^ = 0.47) indicating that P3 was smaller in the prelingually deaf patients (7.536 μV/m^2^ ± 2.35) than the control participant (23.495 μV/m^2^ ± 2.44). However, as with the accuracy data, there was an interaction between “SCD interval × group” (F(1,25) = 3.924; p = 0.05; η_p_^2^ = 0.136) showing the group differences for P3 ERP in SCD0 condition (t(25) = −4.522; p < 0.001) and the SCD300 condition (t(25) = −3.929; p = 0.001). As with the P1 and N1 data, another baseline (i.e. from −200 to 0), or a peak-to-peak quantification procedure of the ERPs did not change the pattern of results. The analysis of the SNR-data revealed no main or interaction effects (all F < 0.9; p > 0.4), showing that the results obtained are unbiased with respect the SNR. The inclusion of “age” as covariate did not change the pattern of results (all F < 0.15; p > 0.4). For the SCD0 condition, the sLORETA analysis revealed that differences between controls and prelingually deaf people (controls>prelingual deafs) are due to the left inferior parietal cortex (BA40) including the temporo-parietal junction (TPJ). For the SCD300 condition the sLORETA analysis again revealed the left TPJ (BA40) including the supramarginal gyrus and the precuneus (controls > prelingual deafs). The Pearson’s bivariate correlations between the “age of cochlear implant” in the prelingually deaf people and significant P3 differences in the “SCD0” and “SCD300” conditions revealed no correlation (r < −0.466, p > 0.19).

## Discussion

In the current study we examined how far changes in the neural processing architecture caused by sensory deprivation, as in this case, related to prelingual deafness can modulate action cascading processes. Previous research suggests that perceptual and attentional processes modulate action cascading^12^, however, it has remained elusive to what extent changes in the neural architecture due to sensory deprivation may affect complex sensorimotor processes during action cascading. To examine this, we investigated action cascading in prelingually deaf patients as a model of altered sensory processing architecture. The results show that changes in the neural processing architecture, known to be evident in (prelingual) deaf people^15^,^16^,^17^,^18^ and can affect action cascading and hence a major sensorimotor and executive control functions as well.

In particular, the behavioral results show that accuracy of action cascading differed between groups. No group differences were observed for the RT data. Prelingually deaf people showed a higher accuracy in action cascading, especially in the more demanding SCD0 condition, but not in the SCD300 condition. The SCD0 condition is more demanding and effortful, because stopping and changing processes are signaled at the same time. A similar group-dependent modulation was not found for the RT data, which indicates that the differential modulation of accuracy across different levels of task difficulty in prelingually deaf people do not reflect a speed-accuracy trade-off. The neurophysiological data provides insights into the processing stage that is differentially modulated across groups and is associated with the behavioral effects. Generally, the SNR of the neurophysiological signals was comparable between groups showing that the neurophysiological data can reliably be interpreted. Also, age did not affect the results obtained.

On the neurophysiological level, the differential effect observed in accuracy data was reflected in the P3 event-related potential. The P3 ERP reflects a link between stimulus processing and the response selection^30^,^34^,^35^,^36^. In particular, the P3 has been related to the “decision” processes between stimulus evaluation and response selection^30^,^34^,^35^, which is related to the allocation of processing resources^36^. The P3 ERP was higher in controls than in prelingually deaf patients in the SCD0 condition. In fact, the finding that a smaller P3 is related to showing a better behavioral performance has been shown in a number of studies^3^,^4^,^5^. The P3 component has been suggested to reflect processing at a capacity-limited strategic bottleneck^37^,^38^. The likely reason that a higher P3 is found in a group showing compromised behavioral performance (compared to another group) is that the more participants put an effort to simultaneously process the ‘stop-goal’ and the ‘change-goal’, the stronger the interference between these goals becomes^31^. As a result, inhibitory control processes necessary to manage the stopping of a response are apt to intensify. Such increases of response inhibition efforts have been shown to correlate with the higher P3 amplitudes^33^. Activation differences in the TPJ found between prelingually deaf and control subjects that were associated with this P3 amplitude effect are well in line with the interpretation that response selection processes are modulated in prelingually deaf people. The TPJ has previously been reported to be related to modulations in the P3 component^39^ and the TPJ is involved in the chaining of actions^3^,^40^,^41^,^42^ to sustain executive controls^43^. The results suggest that prelingually deaf people show more accurate action cascading due to more efficient neuronal processes related to action selection in the TPJ. However, the TPJ does not only play a role in response selection, but also in sensory processing and integration^24^,^44^,^45^. The TPJ is therefore expected to show changed functional characteristics in a sensory deprived neuronal system as found in prelingually deaf patients. It may be speculated that it is this dual role of the TPJ that makes it possible why sensory deprivation can have an effect on response selection processes and hence on mechanisms downstream the processing cascade of sensory information. The TPJ may consist of overlapping networks important for the processing of sensory information and important for the selection of responses. However, in the SCD300 condition there were changes in neurophysiological responses (i.e. P3 amplitude) also related to parietal regions (supramarginal gyrus) that were not reflected by the behavior. These findings suggest that neurophysiological processes in parietal association cortices are generally altered in prelingually deaf patients but do not always result in overt changes of behavior.

Thus far, the results suggest that changes in the neural processing architecture as observed in prelingually deaf patients affect response selection processes in parietal association cortices. The neurophysiological data suggest that this effect is specific to response selection processes and is unlikely to be a result of altered perceptual gating (P1) and attentional selection (N1) processes. This is because neither the P1, nor the N1 revealed interactive effects of SCD interval and group. Thus, they are not in line with the behavioral data and can therefore not explain behavioral differences. There were only main effects of SCD interval and group. The P1 and the N1 were generally larger in the SCD0 than in the SCD300 condition, because in the SCD0 condition two visual stimuli (i.e., STOP and CHANGE stimuli) occur simultaneously^12^. The group differences observed for the P1 (i.e. smaller P1 in prelingually deaf patient) are likely due to activation differences in the middle occipital gyrus (BA19). In light of generally slowed RTs in prelingually deaf patients, this may suggest that perceptual gating processes are less efficient in prelingually deaf patients. The neurophysiological data on perceptual gating and attentional selection underlines that the processes potentially leading to enhanced action cascading performance are confined to later (response selection) processing stages.

An interesting follow-up research question is whether these effects are confined to early, prelingual deafness. For this group of participants we know that deprivation induces major cortical reorganization processes^46^. However, postlingual deaf adults usually profit from inner ear prostheses (cochlear implants) their hearing abilities are still limited and give rise to altered cognitive processing. Thus, it would be noteworthy to examine whether the modulation of action cascading processes is confined to early reorganization processes or whether restrictions of a sensory system occurring in adulthood also affect response selection processes.

In summary, the results show that changes in neural processing architecture as found in people with sensory deprivation are associated with improvements in action cascading. The results show that this improvement is most probably not due to changes at the perceptual (P1-ERP) and attentional processing level (N1-ERP), but at the response selection level (P3-ERP). Effects of sensory deprivation thus affect processing stages downstream of sensory processing and only these seem to be important for behavioral improvements in situations requiring complex sensorimotor processes and action cascading. It seems that the temporo-parietal junction (TPJ) is important for these effects to occur, possibly because the TPJ may consist of overlapping networks relevant for the processing of sensory information and important for the selection of responses. The results underline the significance of the sensory processing architecture for subsequent response selection processes.

## Materials and Methods

### Participants

Our sample consisted of n = 14 prelingually deaf patients (8 females; mean age = 35.36 ± 13.09) and n = 13 healthy participants (7 females; mean age = 33.85 ± 12.85). The subjects groups did not differ in their age (p > 0.5). Criteria for prelingual deafness were a congenital bilateral profound hearing loss or an onset of profound hearing impairment during early childhood followed by a hearing aid supply within this period. Further criteria included impaired language production skills regarding articulation and phonation. In all prelingual subjects, a cochlear implantation was conducted in adulthood (mean age at implantation 32 ± 14.17). The mean time of aural deprivation between estimated onset of severe hearing loss and cochlear implantation was 29 years (±12.42 years). During the course of the experiment these implants were removed to avoid noise and artifacts in EEG signals and as a result these patients had no hearing ability. All of the participants stated to be right-handed and to have no history of psychiatric or neurologic diseases. The Beck Depression Inventory was used (BDI;^47^,^48^,^49^) to assess the level of depressive symptoms and no differences were found between the prelingually deaf patients (3.64 ± 3.41) and the control participants (3.30 ± 2.32) (p > 0.7). Moreover, there was no difference in the years of education between the groups (p > 0.2). All participants had normal or correct-to-normal vision. All participants were naïve to the experimental design. After the experiment, each participants was reimbursed with 20 Euros. All subjects gave written informed consent. The study was approved by the ethics committee of the medical faculty of the Technische Universität Dresden. The methods were carried out in accordance with the approved guidelines.

### Task

**Figure 4:**
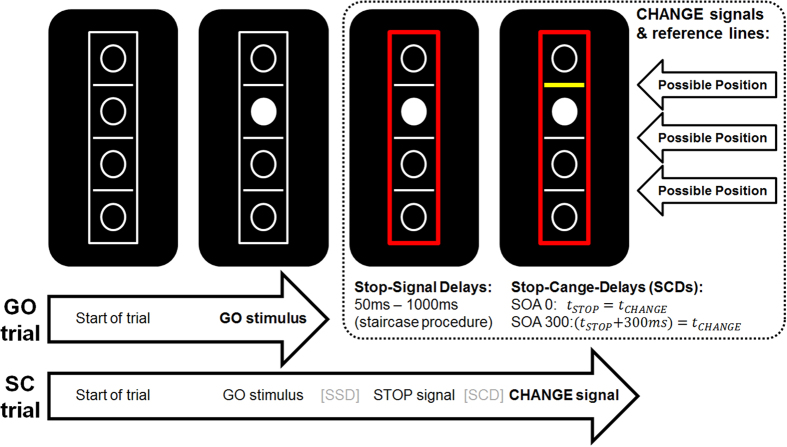
Illustration of the visual stop-change task (SCT). The experiment presented a visual GO signal (white circle) at the beginning of all trials. In GO trials, the subjects needed to respond with the right hand (middle finger “above” response, index finger “below” response). In stop-change trials, the GO stimulus was followed by a visual STOP stimulus (red rectangle, see middle) after a variable and individually adjusted stop-signal delay (SSD). The CHANGE stimulus was either presented with a stimulus onset asynchrony (SOA)/stop-signal delay (SCD) of 0 ms or of 300 ms after the STOP stimulus. The CHANGE stimulus was a bold yellow line. Responses to the CHANGE stimulus had to be given with the left hand (middle finger “above” response, index finger “below” response).

The experimental paradigm (i.e., stop-change paradigm) is shown in Fig. 4.

It was adapted from^32^ and identical to^12^. It was a purely visual stop-change paradigm. The whole experiment lasted approx. 25 minutes. The experiment was conducted in a sound-attenuated room. Each subject was comfortably seated at a distance of 56.5 cm from a 21 inch computer monitor. A custom-made keyboard with four different keys was placed in front of the participants to record the responses. The presentation of experimental stimuli, the recording of behavioral responses and triggering of the EEG were attained by using “Presentation” software (Neurobehavioral Systems, Inc.). In total 864 trials (divided into six blocks) were presented during the experiment.

Out of these trials, 66% were GO trials and 33% were stop-change (SC) trials. All trials were presented in a pseudo-randomized order to avoid preparatory effects in the motor system. Stimuli were presented against a black background. The task array consisted of 4 vertically arranged, white-bordered circles separated by 3 white horizontal bars (reference lines), which were enclosed in a white-bordered rectangle (as shown in Fig. 4). Each trial began with this empty array. After 250 ms one of the four circles was filled in with white color. In GO trails, this white circle became the target and participants had to respond with the right hand: In case the target was located above the middle reference line, participants had to respond with their right middle finger and had to respond with their right index finger if the target was located below that line. If participants did not respond within 1000 ms after the onset of the target, a speed-up sign (containing the German word “Schneller!” translating to “Faster!”) was presented until the trial was ended by a button press.

Stop-change (SC) trials also began with the empty array followed by the GO stimulus, but after a variable stop signal delay (SSD), the GO stimulus was followed by a STOP stimulus. As a STOP stimulus the border of the rectangle turned from white to red (see Fig. 1) and participants had to stop (interrupt) the already initiated right hand GO response. The stop stimulus was always followed by a CHANGE stimulus in two conditions: In the first condition, there was no delay between the STOP and the CHANGE stimuli (i.e., a stop-change delay of 0 ms, called SCD0). In the second condition, there was a stop-change delay of 300 ms (SCD300) so that the CHANGE stimulus was presented 300 ms after the onset of the STOP stimulus. The change stimuli were yellow bars, which remained on the screen until the participant responded by pressing one of the response keys. In each SC trial, one of the three horizontal lines would turn into a thick yellow bar, thus becoming the new reference line that needed to be attended. The participants were asked to spatially relate the target (white circle) to the new reference line. In case the target was located above the yellow reference line, participants had to respond with their left middle finger. When the target was located below the reference line participants had to respond with their left index finger. In case participants did not respond within 2000 ms after the onset of the CHANGE stimulus, the speed-up sign was presented above the stimulus array until the trial was ended by a button press. The SSD described above was initially set to 250 ms and dynamically adjusted to the performance by means of a staircase algorithm^32^. When the participant did not make any mistakes during an SC trial (i.e., did not respond before the presentation of the STOP stimulus and correctly responded to the CHANGE stimulus), the SSD for the following SC trial was increased by 50 ms. In case of any incorrect response, the SSD was decreased by 50 ms. Hence, the staircase yielded a 50% probability of successful inhibition upon stop signal presentation. To keep the trial duration within reasonable limits, SSD variation was restricted to a range from 50 to 1000 ms. On the basis of the assumptions of the horse-race model, the stop signal reaction time (SSRT) can be calculated by subtracting mean SSD from the untrimmed mean GO RT^32^.

### EEG recording and analysis

High-density EEG recording was acquired using 60 Ag–AgCl electrodes at standard scalp positions in an equidistant electrode setup (Quick-Amp amplifier, Brain Products, Inc.). The reference electrode during recording was located at electrode Fpz. The data were recorded with 1 kHz and then down-sampled offline to 256 Hz. All electrode impedances were kept below 5 kΩ. Afterwards, an IIR band-pass filter ranging from 0.5 to 20 Hz. A manual inspection of the data was performed to remove technical artifacts. To correct the periodically recurring artifacts such as pulse, eye blinks or saccade artifacts, an independent component analysis (ICA) was applied using the infomax algorithm. Afterwards, the EEG data was segmented according to the two SCD conditions (i.e. SCD0 and SCD300). Only correct trials were included in the data analysis, i.e. the GO response was successfully stopped and the response on the CHANGE stimulus was also correct. The segmentation was performed in relation to the occurrence of the stop signal^50^; i.e. time point zero in the epochs was set to the occurrence of the STOP stimulus. This was done for the SCD0 and SCD300 condition (e.g.^3^. After the data were epoched, an automated artifact rejection was applied. The rejection criteria included a voltage of more than 150 μV/ms, a value difference of more than 150 μV in a 250-ms interval, or activity below 0.1 μV in a 100 ms interval. This artifact rejection procedure eliminated approx. 8% of trials in each group (controls: 7.99 ± 0.82; patients: 8.23 ± 1.1; p > 0.4). To eliminate the reference potential from the data, a current source density (CSD) transformation was run^51^. In addition to removing the reference potential, the CSD serves as a spatial filter^51^. This helps to identify the electrodes that best reflect activity related to cognitive processes. Thereafter, and prior averaging, a baseline correction was made within the time window from −900 to −700 ms. The baseline was not set prior to the presentation of the stop stimulus, since such a baseline is biased by activity related to the processing of the GO stimulus^3^.

Next, the P1, N1, and P3 event-related potentials (ERPs) were quantified. Electrodes were chosen on the basis of the scalp topographies by visual inspection. The mean amplitudes for the visual P1 and N1 were quantified at electrodes P7 and P8 for the STOP and CHANGE stimuli (P1: SCD0 STOP/CHANGE: 100–140 ms and SCD300 STOP: 100–140 ms/SCD300 CHANGE: 400–450 ms; N1: SCD0 STOP/CHANGE: 200–240 ms and SCD300 STOP: 200–250 ms/SCD300 CHANGE: 520–560 ms post-stimulus, respectively), and the mean amplitudes for the P3 was quantified at Cz (SCD0: 310–350 ms and SCD300: 290–410 ms). This choice of electrodes was validated by a procedure described in^3^: In this procedure, each electrode is compared against an average of all other electrodes using Bonferroni-correction for multiple comparisons (critical threshold p = 0.0007). Only electrodes that showed significantly larger mean amplitudes (i.e., negative for N-potentials and positive for the P- potentials) than the remaining electrodes were chosen. This validation procedure revealed the same electrode positions. All ERP components were quantified relative to the pre-stimulus baseline. For all components, we quantified peak amplitude and latency on the single-subject level. In order to obtain an estimate about the reliability of the neurophysiological data in the groups we calculate the signal-to-noise (SNR) in the prelingual deaf patients and controls as implemented in the Brain Vision Analyzer II software package (BrainProducts Inc.).

### Source localization analysis

Source localization was conducted using sLORETA (standardized low resolution brain electromagnetic tomography^52^. sLORETA gives a single linear solution to the inverse problem, based on extra-cranial measurements without a localization bias^52^,^53^,^54^. It has been mathematically proven that sLORETA provides reliable results without localization bias^54^ and there is evidence of EEG/fMRI and EEG/TMS studies underlining the validity of the sources estimated using sLORETA^31^,^54^. For sLORETA, the intracerebral volume is partitioned into 6239 voxels at 5 mm spatial resolution. The standardized current density at each voxel is calculated in a realistic head model^55^ using the MNI152 template^56^. In this study, the voxel-based sLORETA images were compared across groups (i.e., prelingually deaf vs. control subjects) using the sLORETA-built-in voxel-wise randomization tests with 2000 permutations, based on statistical nonparametric mapping (SnPM). Voxels with significant differences (p < 0.01, corrected for multiple comparisons) between contrasted groups were located in the MNI-brain www.nizh.ch/keyinst/NewLORETA/sLORETA/sLORETA.htm

### Statistics

For all statistics the mean and standard error of the mean (SEM) are given. Behavioral and neurophysiological data (ERP data) were analyzed using mixed effects analyses of variance (ANOVAs). The factors “condition” (SCD0 and SCD300) and “electrode” (only for ERP data) were used as within-subject factors. The factor “group” (prelingually deaf patients vs. controls) was used as a between-subjects factor. The degrees of freedom were adjusted accordingly using Greenhouse-Geisser correction. All post hoc tests were Bonferroni-corrected. Kolmogorov–Smirnov tests indicated that all variables used for the analysis were normally distributed (all z < 0.8; p > 0.6). As the age range in the patients and control sample was rather broad, we included age as covariate in the analyses to control for the effect of age on results obtained.

## Additional Information

**How to cite this article**: Gohil, K. *et al*. Improvements of sensorimotor processes during action cascading associated with changes in sensory processing architecture–insights from sensory deprivation. *Sci. Rep.* **6**, 28259; doi: 10.1038/srep28259 (2016).

## Supplementary Material

Supplementary Information

## References

[b1] Duncan J. The multiple-demand (MD) system of the primate brain: mental programs for intelligent behaviour. Trends in Cognitive Sciences 14, 172–179 (2010). 20171926 10.1016/j.tics.2010.01.004

[b2] Mückschel M., Stock A.-K. & Beste C. Different strategies, but indifferent strategy adaptation during action cascading. Sci. Rep. 5, 9992 (2015). 25950375 10.1038/srep09992 PMC4649999

[b3] Mückschel M., Stock A.-K. & Beste C. Psychophysiological mechanisms of interindividual differences in goal activation modes during action cascading. Cereb. Cortex N. Y. N 1991 24, 2120–2129 (2014). 10.1093/cercor/bht066 23492952

[b4] Stock A.-K., Arning L., Epplen J. T. & Beste C. DRD1 and DRD2 genotypes modulate processing modes of goal activation processes during action cascading. J. Neurosci. Off. J. Soc. Neurosci. 34, 5335–41 (2014). 10.1523/JNEUROSCI.5140-13.2014 PMC6608997 24719111

[b5] Beste C., Stock A.-K., Epplen J. T. & Arning L. On the relevance of the NPY2-receptor variation for modes of action cascading processes. NeuroImage 102, 558–564 (2014). 25157429 10.1016/j.neuroimage.2014.08.026

[b6] Beste C., Kneiphof J. & Woitalla D. Modulatory effects of proinflammatory cytokines for action cascading processes-evidence from neurosarcoidosis. Brain. Behav. Immun. 41, 126–33 (2014). 24846477 10.1016/j.bbi.2014.05.005

[b7] Beste C. & Saft C. Action selection in a possible model of striatal medium spiny neuron dysfunction: behavioral and EEG data in a patient with benign hereditary chorea. Brain Struct. Funct. 10.1007/s00429-013-0649-9 (2013). 24135770

[b8] Stock A.-K., Gohil K. & Beste C. Age-related differences in task goal processing strategies during action cascading. Brain Struct. Funct. 10.1007/s00429-015-1071-2 (2015). 26025200

[b9] Yildiz A., Wolf O. T. & Beste C. Stress intensifies demands on response selection during action cascading processes. Psychoneuroendocrinology 42, 178–187 (2014). 24636514 10.1016/j.psyneuen.2014.01.022

[b10] Soto-Faraco S. & Spence C. Modality-specific auditory and visual temporal processing deficits. Q J Exp Psychol A 55, 23–40 (2002). 11873849 10.1080/02724980143000136

[b11] Gohil K., Stock A.-K. & Beste C. The importance of sensory integration processes for action cascading. Sci. Rep. 5, (2015). 10.1038/srep09485 PMC4377632 25820681

[b12] Gohil K., Stock A.-K. & Beste C. The importance of sensory integration processes for action cascading. Sci. Rep. 5, 9485 (2015). 25820681 10.1038/srep09485 PMC4377632

[b13] Dehaene S., Kerszberg M. & Changeux J. P. A neuronal model of a global workspace in effortful cognitive tasks. Proc. Natl. Acad. Sci. USA 95, 14529–14534 (1998). 9826734 10.1073/pnas.95.24.14529 PMC24407

[b14] Ding H. . Cross-modal activation of auditory regions during visuo-spatial working memory in early deafness. Brain J. Neurol. 10.1093/brain/awv165 (2015). 26070981

[b15] Finney E. M. & Dobkins K. R. Visual contrast sensitivity in deaf versus hearing populations: Exploring the perceptual consequences of auditory deprivation and experience with a visual language. Cogn. Brain Res. 11, 171–183 (2001). 10.1016/s0926-6410(00)00082-3 11240120

[b16] Lomber S. G., Meredith M. A. & Kral A. Cross-modal plasticity in specific auditory cortices underlies visual compensations in the deaf. Nat. Neurosci. 13, 1421–7 (2010). 20935644 10.1038/nn.2653

[b17] Meredith M. A. . Crossmodal reorganization in the early deaf switches sensory, but not behavioral roles of auditory cortex. Proc. Natl. Acad. Sci. USA 108, 8856–61 (2011). 21555555 10.1073/pnas.1018519108 PMC3102418

[b18] Karns C. M., Dow M. W. & Neville H. J. Altered cross-modal processing in the primary auditory cortex of congenitally deaf adults: a visual-somatosensory fMRI study with a double-flash illusion. J. Neurosci. Off. J. Soc. Neurosci. 32, 9626–38 (2012). 10.1523/JNEUROSCI.6488-11.2012 PMC3752073 22787048

[b19] Röder B. . Improved auditory spatial tuning in blind humans. Nature 400, 162–166 (1999). 10408442 10.1038/22106

[b20] Van Boven R. W., Hamilton R. H., Kauffman T., Keenan J. P. & Pascual-Leone A. Tactile spatial resolution in blind braille readers. Neurology 54, 2230–6 (2000). 10881245 10.1212/wnl.54.12.2230

[b21] Gougoux F., Zatorre R. J., Lassonde M., Voss P. & Lepore F. A functional neuroimaging study of sound localization: visual cortex activity predicts performance in early-blind individuals. PloS Biol. 3, e27 (2005). 15678166 10.1371/journal.pbio.0030027 PMC544927

[b22] Dye M. W. G., Hauser P. C. & Bavelier D. Is visual selective attention in deaf individuals enhanced or deficient? The case of the useful field of view. PloS One 4, e5640 (2009). 19462009 10.1371/journal.pone.0005640 PMC2680667

[b23] Yildiz A. . Feeling safe in the plane: Neural mechanisms underlying superior action control in airplane pilot trainees-A combined {EEG/MRS} study. Hum Brain Mapp 35, 5040–5051 (2014). 24753040 10.1002/hbm.22530 PMC4452896

[b24] Ionta S. . Multisensory mechanisms in temporo-parietal cortex support self-location and first-person perspective. Neuron 70, 363–74 (2011). 21521620 10.1016/j.neuron.2011.03.009

[b25] Matsuhashi M. . Multisensory convergence at human temporo-parietal junction-epicortical recording of evoked responses. Clin. Neurophysiol. Off. J. Int. Fed. Clin. Neurophysiol. 115, 1145–60 (2004). 10.1016/j.clinph.2003.12.009 15066540

[b26] Luck S., Woodman G. & Vogel E. Event-related potential studies of attention. Trends Cogn. Sci. 4, 432–440 (2000). 11058821 10.1016/s1364-6613(00)01545-x

[b27] Wascher E. & Beste C. Tuning perceptual competition. J. Neurophysiol. 103, 1057–65 (2010). 20032246 10.1152/jn.00376.2009

[b28] Hopf J. M., Boelmans K., Schoenfeld A. M., Heinze H. J. & Luck S. J. How does attention attenuate target-distractor interference in vision? Evidence from magnetoencephalographic recordings. Cogn. Brain Res. 15, 17–29 (2002). 10.1016/s0926-6410(02)00213-6 12433380

[b29] Vogel E. K. & Luck S. J. The visual N1 component as an index of a discrimination process. Psychophysiology 37, 190–203 (2000). 10731769

[b30] Verleger R., Jaśkowski P. & Wascher E. Evidence for an integrative role of P3b in linking reaction to perception. J. Psychophysiol. 19, 165–181 (2005).

[b31] Dippel G. & Beste C. A causal role of the right inferior frontal cortex in the strategies of multi-component behaviour. Nat. Commun. 6, 6587 (2015). 25850926 10.1038/ncomms7587

[b32] Verbruggen F., Schneider D. W. & Logan G. D. How to stop and change a response: the role of goal activation in multitasking. J. Exp. Psychol. Hum. Percept. Perform. 34, 1212–1228 (2008). 18823206 10.1037/0096-1523.34.5.1212

[b33] Huster R. J., Enriquez-Geppert S., Lavallee C. F., Falkenstein M. & Herrmann C. S. Electroencephalography of response inhibition tasks: functional networks and cognitive contributions. Int. J. Psychophysiol. Off. J. Int. Organ. Psychophysiol. 87, 217–33 (2013). 10.1016/j.ijpsycho.2012.08.001 22906815

[b34] Falkenstein M., Hohnsbein J. & Hoormann J. Time pressure effect on late components of the event-related potential (ERP). J. Psychophysiol. 8, 22–30 (1994).

[b35] Falkenstein M., Hohnsbein J. & Hoormann J. Effects of choice complexity on different subcomponents of the late positive complex of the event-related potential. Electroencephalogr. Clin. Neurophysiol. 92, 148–160 (1994). 7511512 10.1016/0168-5597(94)90055-8

[b36] Polich J. Updating P300: an integrative theory of P3a and P3b. Clin. Neurophysiol. Off. J. Int. Fed. Clin. Neurophysiol. 118, 2128–48 (2007). 10.1016/j.clinph.2007.04.019 PMC2715154 17573239

[b37] Sigman M. & Dehaene S. Brain mechanisms of serial and parallel processing during dual-task performance. J. Neurosci. Off. J. Soc. Neurosci. 28, 7585–7598 (2008). 10.1523/JNEUROSCI.0948-08.2008 PMC6670853 18650336

[b38] Brisson B. & Jolicoeur P. Cross-modal multitasking processing deficits prior to the central bottleneck revealed by event-related potentials. Neuropsychologia 45, 3038–3053 (2007). 17659310 10.1016/j.neuropsychologia.2007.05.022

[b39] Verleger R., Heide W., Butt C. & Kömpf D. Reduction of P3b in patients with temporo-parietal lesions. Brain Res. Cogn. Brain Res. 2, 103–16 (1994). 7833690 10.1016/0926-6410(94)90007-8

[b40] Chersi F., Ferrari P. F. & Fogassi L. Neuronal chains for actions in the parietal lobe: a computational model. PloS One 6, e27652 (2011). 22140455 10.1371/journal.pone.0027652 PMC3225358

[b41] Karch S. . Separating distinct aspects of the voluntary selection between response alternatives: N2- and P3-related BOLD responses. NeuroImage 51, 356–64 (2010). 20171291 10.1016/j.neuroimage.2010.02.028

[b42] Astafiev S. V., Shulman G. L. & Corbetta M. Visuospatial reorienting signals in the human temporo-parietal junction are independent of response selection. Eur. J. Neurosci. 23, 591–6 (2006). 16420468 10.1111/j.1460-9568.2005.04573.x

[b43] Collette F. . Involvement of both prefrontal and inferior parietal cortex in dual-task performance. Brain Res. Cogn. Brain Res. 24, 237–51 (2005). 15993762 10.1016/j.cogbrainres.2005.01.023

[b44] Parker Jones, ’ōiwi . Sensory-to-motor integration during auditory repetition: a combined fMRI and lesion study. Front. Hum. Neurosci. 8, 24 (2014). 24550807 10.3389/fnhum.2014.00024 PMC3908611

[b45] Jakobs O. . Across-study and within-subject functional connectivity of a right temporo-parietal junction subregion involved in stimulus-context integration. NeuroImage 60, 2389–98 (2012). 22387170 10.1016/j.neuroimage.2012.02.037 PMC3321133

[b46] Kral A. & Sharma A. Developmental neuroplasticity after cochlear implantation. Trends Neurosci. 35, 111–122 (2012). 22104561 10.1016/j.tins.2011.09.004 PMC3561718

[b47] Beck A. T., Ward C. H., Mendelson M., Mock J. & Erbaugh J. An inventory for measuring depression. Arch. Gen. Psychiatry 4, 561–71 (1961). 13688369 10.1001/archpsyc.1961.01710120031004

[b48] Rossi S., Hallett M., Rossini P. M. & Pascual-Leone A. Screening questionnaire before TMS: an update. Clin. Neurophysiol. Off. J. Int. Fed. Clin. Neurophysiol. 122, 1686 (2011). 10.1016/j.clinph.2010.12.037 21227747

[b49] Rossi S., Hallett M., Rossini P. M. & Pascual-Leone A. Safety, ethical considerations, and application guidelines for the use of transcranial magnetic stimulation in clinical practice and research. Clin. Neurophysiol. Off. J. Int. Fed. Clin. Neurophysiol. 120, 2008–2039 (2009). 10.1016/j.clinph.2009.08.016 PMC3260536 19833552

[b50] Mückschel M., Stock A.-K. & Beste C. Different strategies, but indifferent strategy adaptation during action cascading. Sci. Rep. 5, 9992 (2015). 25950375 10.1038/srep09992 PMC4649999

[b51] Nunez P. L. & Pilgreen K. L. The spline-Laplacian in clinical neurophysiology: a method to improve EEG spatial resolution. J. Clin. Neurophysiol. Off. Publ. Am. Electroencephalogr. Soc. 8, 397–413 (1991). 1761706

[b52] Pascual-Marqui R. D., Esslen M., Kochi K. & Lehmann D. Functional imaging with low-resolution brain electromagnetic tomography (LORETA): a review. Methods Find. Exp. Clin. Pharmacol. 24 Suppl C, 91–5 (2002). 12575492

[b53] Marco-Pallarés J., Grau C. & Ruffini G. Combined ICA-LORETA analysis of mismatch negativity. NeuroImage 25, 471–7 (2005). 15784426 10.1016/j.neuroimage.2004.11.028

[b54] Sekihara K., Sahani M. & Nagarajan S. S. Localization bias and spatial resolution of adaptive and non-adaptive spatial filters for MEG source reconstruction. NeuroImage 25, 1056–67 (2005). 15850724 10.1016/j.neuroimage.2004.11.051 PMC4060617

[b55] Fuchs M., Kastner J., Wagner M., Hawes S. & Ebersole J. S. A standardized boundary element method volume conductor model. Clin. Neurophysiol. Off. J. Int. Fed. Clin. Neurophysiol. 113, 702–12 (2002). 10.1016/s1388-2457(02)00030-5 11976050

[b56] Mazziotta J. . A probabilistic atlas and reference system for the human brain: International Consortium for Brain Mapping (ICBM). Philos. Trans. R. Soc. Lond. B. Biol. Sci. 356, 1293–322 (2001). 11545704 10.1098/rstb.2001.0915 PMC1088516

