# Supplementary material for: Improvements of sensorimotor processes during action cascading associated with changes in sensory processing architecture–insights from sensory deprivation
Source: Sci Rep. 2016 Jun 20;6:28259. doi: 10.1038/srep28259 (PMC4913291; doi:10.1038/srep28259)
Supplement: Supplementary Information [file srep28259-s1.pdf]

Improvements of sensorimotor processes during action cascading associated with changes in sensory processing architecture – insights from sensory deprivation

Krutika Gohil[1], Anja Hahne[2], Christian Beste[1,*]

[1] Cognitive Neurophysiology, Department of Child and Adolescent Psychiatry, Faculty of Medicine of the TU Dresden, Germany
[2] Saxonian Cochlear Implant Center, Division of Phoniatrics and Audiology, Department of Otorhinolaryngology, Faculty of Medicine of the TU Dresden, Germany

Supplementary Table S1

Descriptive behavioral data for the control group and the prelingual deaf patient group. The mean and standard deviation are given.

	**Control group**	**Prelingual deaf patient group**
Go accuracy (number of correct trials)	540.76 (6.42)	557.5 (6.18)
SCD0 accuracy (number of correct trials)	73.58 (5.02)	99.35 (4.84)
SCD300 accuracy (number of correct trials)	116.75 (5.87)	126.28 (5.66)
Go RTs	519ms (67)	703ms (65)
SCD0 RTs	727ms (45)	933ms (43)
SCD300 RTs	559ms (45)	776ms (43)
SSRT	444ms (65)	882ms (62)
SSD	274ms (28)	320ms (18)
